# In Vivo Bioimpedance Spectroscopy Characterization of Healthy, Hemorrhagic and Ischemic Rabbit Brain within 10 Hz–1 MHz

**DOI:** 10.3390/s17040791

**Published:** 2017-04-07

**Authors:** Lin Yang, Wenbo Liu, Rongqing Chen, Ge Zhang, Weichen Li, Feng Fu, Xiuzhen Dong

**Affiliations:** 1 Department of Biomedical Engineering, Fourth Military Medical University, Xi’an 710032, China; yanglin.0601@163.com (L.Y.); andreaschan@foxmail.com (R.C.); jsj.202@163.com (G.Z.); oliver2015@fmmu.edu.cn (W.L.); 2 Department of Neurosurgery, Xijing hospital, Fourth Military Medical University, Xi’an 710032, China; boboliucn@126.com

**Keywords:** brain impedance spectra, rabbits, stroke

## Abstract

Acute stroke is a serious cerebrovascular disease and has been the second leading cause of death worldwide. Conventional diagnostic modalities for stroke, such as CT and MRI, may not be available in emergency settings. Hence, it is imperative to develop a portable tool to diagnose stroke in a timely manner. Since there are differences in impedance spectra between normal, hemorrhagic and ischemic brain tissues, multi-frequency electrical impedance tomography (MFEIT) shows great promise in detecting stroke. Measuring the impedance spectra of healthy, hemorrhagic and ischemic brain in vivo is crucial to the success of MFEIT. To our knowledge, no research has established hemorrhagic and ischemic brain models in the same animal and comprehensively measured the in vivo impedance spectra of healthy, hemorrhagic and ischemic brain within 10 Hz–1 MHz. In this study, the intracerebral hemorrhage and ischemic models were established in rabbits, and then the impedance spectra of healthy, hemorrhagic and ischemic brain were measured in vivo and compared. The results demonstrated that the impedance spectra differed significantly between healthy and stroke-affected brain (i.e., hemorrhagic or ischemic brain). Moreover, the rate of change in brain impedance following hemorrhagic and ischemic stroke with regard to frequency was distinct. These findings further validate the feasibility of using MFEIT to detect stroke and differentiate stroke types, and provide data supporting for future research.

## 1. Introduction

Acute stroke, a serious and acute cerebrovascular disease, is classified into two clinical types: (1) hemorrhagic stroke, caused by blood bleeding into the brain tissue or the subarachnoid space through a ruptured intracranial vessel, which accounts for 13% of all stroke cases; and (2) ischemic stroke, caused by vascular occlusion in the brain due to a blood clot (thrombosis), which accounts for 87% of all stroke cases [1]. Stroke is characterized by sudden onset and high mortality and has been the second leading cause of death worldwide [2]. Prompt intervention improves the prognosis of stroke patients. Additionally, distinct interventions are needed for each stroke subtype. Hemorrhagic stroke patients require prompt surgical intervention, while ischemic stroke patients need thrombolytic therapy with tissue plasminogen activator within 3–4.5 h after the onset of stroke [3]. Hence, stroke should be typed to prevent the adverse effects of thrombolytic therapy on hemorrhagic stroke patients. Currently, CT and MRI are the main diagnostic tools used to diagnose stroke. However, in an emergency setting (e.g., in an ambulance), it is impractical to image the brain with CT or MRI [4]. Therefore, a portable tool that can detect stroke quickly under these circumstances is clearly needed.

As a type of electrical impedance tomography (EIT), multi-frequency EIT (MFEIT) reconstructs the impedance distribution inside the human body according to the principle that the impedance of a biological tissue changes with frequency. In MFEIT, multi-frequency currents are simultaneously delivered through surface electrodes placed on the human body and the resulting boundary voltages are measured [5]. In this way, tissues are distinguished on basis of their specific impedance spectra [6]. Time-difference EIT (td-EIT) recovers the impedance change over time; but it is difficult to obtain data before disease onset in practice and thus td-EIT cannot be used to detect stroke [7]. Unlike td-EIT, MFEIT does not need any reference or baseline data acquired at other time points. Since there are difference in impedance spectra between normal, hemorrhagic and ischemic brain tissues [8], MFEIT shows promise in becoming an imaging modality that can quickly detect stroke and can also be used to identify stroke subtypes [4,9,10,11].

Previously, Yang et al. established brain hemorrhagic and ischemic models in rabbits and measured the impedance spectra of normal, hemorrhagic and ischemic brain tissues ex vivo [8]. These accurate tissue impedance spectral data form an important basis for stroke detection with MFEIT; however, these data may not fully reflect the features of impedance spectra of the healthy, hemorrhagic and ischemic brain when measured in vivo. Essentially, it is important to measure these features in vivo for MFEIT to be successful in detecting stroke, because MFEIT uses these features to image tissues [12,13]. In particular, the impedance spectra from the same animal species allow direct comparison of healthy, hemorrhagic and ischemic brain. To date, several research groups have investigated the impedance spectra of the healthy, hemorrhagic and ischemic brain. Lingwood et al. [14] and Ranck [15] measured the in vivo impedance spectra of healthy monkey (10 Hz–5 kHz) and rabbit (5 Hz–50 kHz) brains, respectively. Dowrick et al. made the measurement in vivo in healthy rabbit brains from 10 Hz to 3 kHz [16]. However, these studies did not establish animal models of hemorrhagic and ischemic brain, and measured their impedance spectra. In addition, these studies did not determine the impedance spectra at frequencies above 50 kHz (to improve the performance of MFEIT in detecting stroke, a frequency range of up to 1 MHz was suggested to be used [5,17]).

In the case of ischemic brain, Dowrick et al. established an ischemic model in rats by occluding four vessels, and used four-electrode technique to measure the in vivo impedance spectra of the brain before and after ischemic stroke across the 1 Hz–3 kHz range [16]. Seoane et al. used four-electrode technique to determine the impedance spectra of the brain before and after hypoxia in neonatal and adult pigs across the 20–750 kHz range [18]. Wu et al. established an ischemic brain model in rabbits by ligating the carotid arteries, and used two-electrode technique to measure the impedance spectra of normal and ischemic brain across the 0.1 Hz–1 MHz range [19]. Other research groups monitored changes in impedance before and after brain ischemia at a single frequency [20,21]. Nevertheless, in these studies, the measurements of impedance spectra of hemorrhagic brain were not taken. Moreover, in the study by Wu et al., the two-electrode technique might affect the measurement results because of electrode contact impedance [16,22]. As for the hemorrhagic brain, several research groups monitored changes in the brain impedance before and after hemorrhagic stroke at a single frequency [20,23,24]. Seoane et al. measured and compared the impedance spectra of healthy and stroke-affected human brains across the 3.096–1000 kHz range [25]. However, these studies did not carried out the measurement of brain impedance within a wide frequency band (1 MHz). In the study by Seoane et al., because the results were obtained from different human subjects, the comparison of brain impedance before and after stroke was limited. In conclusion, although a number of existing publications have reported the impedance spectra of healthy, hemorrhagic and ischemic brain, the measurement results cannot be directly compared because of variations in experimental animals, modeling methods and measuring conditions. Therefore, it is essential to establish hemorrhagic and ischemic brain models in the same animal species, and comprehensively measure and compare the impedance spectra of healthy, hemorrhagic and ischemic brain across the 10 Hz–1 MHz range.

In this study, the intracerebral hemorrhage and ischemic models were established in rabbits, and the impedance spectra of healthy, hemorrhagic and ischemic brain were measured in vivo from 10 Hz to 1 MHz. Then, the difference in impedance spectra between healthy and stroke-affected brain was analyzed to assess the feasibility of using MFEIT to detect stroke, and difference in the rate of change of brain impedance between hemorrhagic and ischemic stroke with regard to frequency was also analyzed to identify stroke subtypes. Based on the results, the optimal frequency ranges for MFEIT to detect stroke and identify stroke subtypes were discussed.

## 2. Materials and Methods

### 2.1. Ethical Statement

All animal experiments in this study were approved by the Ethics Committee for Animal Studies of the Fourth Military Medical University, Xi’an, Shaanxi, China.

### 2.2. Preparation of Animals

Forty New Zealand rabbits (2.2 ± 0.3 kg), obtained from the Laboratory Animal Center of the Fourth Military Medical University, were divided into four groups: (1) hemorrhage group (n = 10); (2) hemorrhage control group (n = 10); (3) ischemia group (n = 10); and (4) ischemia control group (n = 10). Rabbits were deprived of food for 4 h and of water for 2 h before all experimental procedures. They were sedated by intraperitoneal injection of 1.5% pentobarbital sodium (2 mL/kg), followed by deep anesthesia by injecting 3% pentobarbital sodium (0.5 mL/kg) into the ear rim vein. During surgery, 1.5% pentobarbital sodium was injected intraperitoneally at a rate of 1 mL·kg^−1^·h^−1^ to keep the animal sedated. Animal body temperature was maintained at 39.5 ± 0.5 °C with a warm water blanket and was measured with a rectal thermistor probe. Each animal was immobilized onto a stereotactic frame in a prone position by using eye- and ear-fixing bars.

### 2.3. Surgery

**Figure 1:**
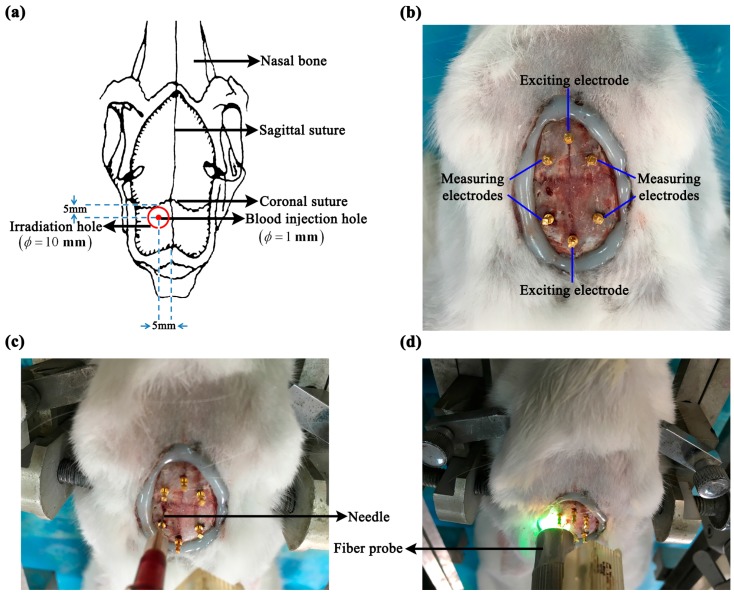
Localized intracerebral hemorrhage and ischemic models. (**a**) Schematic diagram. (**b**) Electrode distribution for in vivo measurement of whole-brain impedance spectra. (**c**,**d**) Intracerebral hemorrhage model (autologous blood injection method) and ischemic model (photochemical induction method).

Because stroke is primarily characterized by localized hemorrhage or ischemia, localized intracerebral hemorrhage and ischemic models were established. The hair on the rabbit’s head (approximately 15 cm^2^) was shaved with electric clippers. Then, the scalp and periosteum were removed with a scalpel; this was followed by electrocoagulation. Once hemostasis was achieved, the surgical field was cleared. The near-elliptical wound was approximately 3.6 cm long in the sagittal direction, and approximately 2.5 cm long in the coronal direction (Figure 1). To minimize water loss from the cranium and wound, an even layer of bone wax was smeared on the exposed cranium and an even layer of medical glue was smeared on the wound surface.

#### 2.3.1. Intracerebral Hemorrhage Model

The intracerebral hemorrhage model was established using autologous blood injection method [24]. The animal was immobilized onto the stereotactic frame and a hole was drilled with a dental bur 5 mm to the left of the sagittal suture and 5 mm behind the coronal suture. The hole was deep into the dura and was 1 mm in diameter (Figure 1a,c). Next, 1 mL of autologous blood was drawn from the heart and 0.4 mL of blood was aspirated into a 1-mL syringe. The syringe was fixed onto the stereotactic frame and the needle was inserted into the cranial hole. According to the anatomy of the rabbit brain, the needle was inserted at a depth of 11 mm to ensure that the blood was injected into the brain parenchyma. The injection of blood was started 1 min after the needle was inserted, and was completed within 2 min. The needle was withdrawn 30 min after completion of the injection. Finally, the rabbit was sacrificed by administering a pentobarbital overdose before the whole brain was taken out to immerse in formaldehyde overnight for 24 h. In the hemorrhage control group, all surgical steps were the same as those used for the hemorrhage group, except for the blood injection.

#### 2.3.2. Ischemic Model

The ischemic model was established using photothrombotic stroke method [26]. A dental bur (10 mm in diameter) was used to drill the cranium 6 mm to the left of the sagittal suture and 5 mm behind the coronal suture through the outer plate layer to expose the inner plate layer, resulting in a 10-mm-wide round hole. Hemostasis was achieved by a gelatin sponge, and then the operating field was cleared (Figure 1a,d). Next, 3.5% rose bengal dye (Sigma-Aldrich Corporation, St Louis, MO, USA) was slowly injected into the ear rim vein at a dose of 1.5 mL/kg. When the rabbit’s eyes turned rose red (normally 15 min after the dye was injected), the LG150B-type cold light source (Photo-Machine Technological Exploration Corporation, Xi’an, Shaanxi, China) was turned on, and pure green light (wavelength: 540 nm; intensity: 600 mW/cm^2^) was transmitted via optical fibers. The probe (10 mm in diameter) connected to the optical fibers was placed perpendicular to the cranial hole, and the cranial hole was irradiated for 30 min. Finally, the rabbit was sacrificed by administrating a pentobarbital overdose and the whole brain was taken out to immerse in formaldehyde overnight for 24 h. In the ischemia control group, all surgical steps were the same as those in the ischemia group, except for green light irradiation.

### 2.4. In Vivo Measurement of Brain Impedance Spectra

#### 2.4.1. Measurement: Protocol and Hardware

Before establishing the intracerebral hemorrhage or ischemic model, six electrodes (copper nails, 0.93 mm in diameter; Hangzhou Westlake Biomaterial Corporation, Hangzhou, China) were placed onto the rabbit’s cranium. Two of the electrodes were placed along the sagittal suture, one 2 cm in front of and the other 1 cm behind the coronal suture, respectively. Two electrodes were placed on each side of the sagittal suture (0.8 cm away from the sagittal suture), the two electrodes on the same side being 0.8 cm apart in the sagittal direction. All electrodes were inserted approximately 1-mm deep to ensure that none of the copper nails penetrated the cranium. After all the electrodes were placed, they were further fixed onto the cranium with glue (DP100 epoxy adhesive; 3M Corporation, Maplewood, MN, USA; see Figure 1). The four-electrode technique was employed to minimize the effect of electrode contact impedance on the measurement. The two electrodes, located along the sagittal suture, were the exciting electrodes; the electrode in front of the coronal suture was the positive exciting electrode and that behind it was the negative exciting electrode. The electrodes on each side of the sagittal suture were the measuring electrodes; those in front of the coronal suture were the positive measuring electrodes and those behind it were the negative measuring electrodes. In the intracerebral hemorrhage group, the brain impedance spectra were measured 1 min after the needle was inserted, and the impedance spectra of post-hemorrhagic stroke were measured 30 min after blood was injected, i.e., 33 min after the needle was inserted. The time points used to measure the brain impedance were the same for the intracerebral hemorrhage group and its control group. In the ischemic model, the brain impedance spectra were measured before green light irradiation (15 min after injection of rose bengal dye) and 30 min after (45 min after injection of rose bengal dye). The time points used to measure brain impedance spectra in the ischemia group and its control group were the same.

In this study, a Solartron 1260 impedance/gain-phase analyzer (Solartron Analytical, Farnborough, UK) with a 1294A impedance interface system was used to measure impedance, and the ZPlot software (Scribner Associates, Inc., Southern Pines, NC, USA) was utilized to control the acquisition of parameters. A 0.2 mA AC RMS signal was used across the two exciting electrodes to sweep from 10 Hz to 1 MHz in 51 steps. The voltage was measured by the two measuring electrodes and the impedance between the two measuring electrodes was calculated. In each measurement of brain impedance, when the measurement of the brain impedance spectra on one side was completed, the measuring electrodes were immediately replaced with the electrodes on the other side to continue the measurement.

#### 2.4.2. Measurement in Saline Solution

The four electrodes (two exciting electrodes and two measuring electrodes) were immersed into a beaker containing 0.03 M/L saline solution, and the impedance of the saline solution was recorded from 10 Hz to 1 MHz. Theoretically, the impedance of saline solution should not change with frequency [27]. Before each experiment, the impedance of saline solution was measured as the control.

### 2.5. Histopathology

To evaluate the pathological changes of stroke-affected brain tissues, the animals were sacrificed with an overdose of pentobarbital sodium after the completion of the impedance measurement and their brains were taken out. In each group, the normal white and gray matter, and the hemorrhagic (the entire blood clot) and ischemic tissues (area surrounding the location of ischemia), all 1 cm in diameter, were removed from the brain and fixed in 10% formaldehyde for 24 h. After fixation, tissue specimens were cut into 3-mm thick sections, stained with hematoxylin and eosin and reviewed by a pathologist.

### 2.6. Data Analysis

The impedance spectra of each side of the brain are denoted by $Z_{L}$ and $Z_{R}$, respectively. The sum of the measurements of both sides ($Z=Z_{L}+Z_{R}$) was used to investigate the impedance spectra of the whole brain. Because measurements were taken across a wide frequency range (10 Hz–1 MHz), the imaginary part of brain impedance was considered, and brain impedance was denoted by $Z=\;Z_{real}+jZ_{imag}$, where $Z_{real}$ and $Z_{imag}$ are the real and imaginary part of brain impedance, respectively; j is the imaginary unit number and $j^{2}=-1$. For the four groups of animals, the brain impedance spectra were measured at two time points respectively denoted by $Z_{before}=Z_{real}^{before}+jZ_{imag}^{before}$ and $Z_{after}=Z_{real}^{after}+jZ_{imag}^{after}$, where $Z_{before}$ and $Z_{after}$ were the first and second measurements respectively.

When using MFEIT to detect stroke, the differential result of data at two different frequencies is preferred to eliminate common data errors, such as unknown boundary geometry and uncertainty regarding electrode position [7,10,28]. Therefore, it is important to know the relative change in brain impedance across the frequency range, rather than the absolute impedance. In this study, 10 Hz was used as the reference frequency to calculate the relative change in brain impedance across the 10 Hz–1 MHz range.

First, to detect stroke with MFEIT, the healthy brain should be distinguished from the stroke-affected brain (whether ischemic or hemorrhagic). Hence, the difference $\Delta Z=\Delta Z_{real}+j\Delta Z_{imag}$ in brain impedance spectra between the healthy and stroke-affected brain was analyzed:

(1) $$\Delta Z_{real}=Z_{real}^{after}-Z_{real}^{before}$$

(2) $$\Delta Z_{imag}=Z_{imag}^{after}-Z_{imag}^{before}$$

SPSS 22 (IBM Software, Armonk, NY, USA) was employed for the statistical analysis. The comparisons of brain impedance difference ($\Delta Z_{real}$ and $\Delta Z_{imag}$) at different frequencies between hemorrhage group and hemorrhage control group or between ischemia group and ischemia control group were carried out with one-way analysis of variance (ANOVA). The post-hoc test was used and *p* < 0.05 was deemed statistically significant.

Second, when detecting the early stages of stroke, it is essential to discriminate the stroke subtype. Because MFEIT uses differences in tissue impedance spectra to image stroke, the difference in the rate of change of brain impedance with regard to frequency between hemorrhagic and ischemic stroke may be helpful to identify the type of stroke involved. In this study, because different surgeries were performed on rabbits to establish the intracerebral hemorrhage and ischemic models, it is meaningless to directly compare the impedance spectra of the ischemic and hemorrhagic brain. To address this issue, the whole frequency range was divided into three subranges and the lowest frequency was selected as the reference frequency in each subrange. The three frequency subranges were low (10 Hz–1 kHz; reference frequency: 10 Hz), intermediate (1 kHz–100 kHz; reference frequency: 1 kHz) and high (100 kHz–1 MHz; reference frequency: 100 kHz). For each subrange, the real and imaginary part of impedance spectra of ΔZ at the reference frequency were compared with those at the other frequencies to analyze the rate of change of ΔZ, as denoted by:

(3) $$(\Delta Z_{real}^{f}-\Delta Z_{real}^{ref})/\Delta Z_{real}^{ref}\text{ and }(\Delta Z_{imag}^{f}-\Delta Z_{imag}^{ref})/\Delta Z_{imag}^{ref}$$

## 3. Results

**Figure 2:**
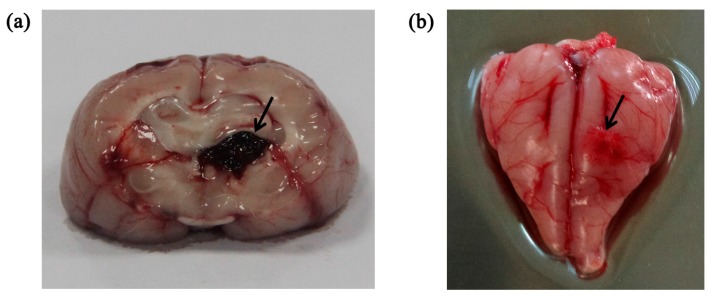
(**a**) Intracerebral hemorrhage model; (**b**) Ischemic model. The arrow indicates the location of the stroke lesion.

In all experiments, the animals had stable body temperature and respiration. Twenty sets of brain impedance spectra were obtained for each of the four study groups. As shown in Figure 2, the site of bleeding was in brain parenchyma (i.e., the white matter) in the intracerebral hemorrhage model and ischemia was mainly located in the cerebral cortex (i.e., the gray matter) in the ischemic model. The volume of the blood clot was approximately 275 ± 25 mm^3^ in the intracerebral hemorrhage group; in the ischemia group, the ischemic tissue was 4.7 ± 0.3 mm in radius and 2.7 ± 0.25 mm in thickness.

### 3.1. Measurement of the Impedance Spectra of Healthy, Hemorrhagic and Ischemic Brain

**Figure 3:**
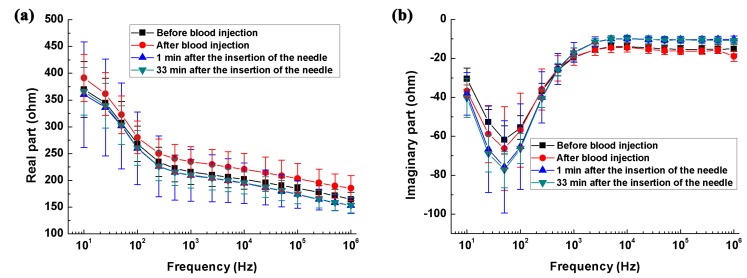

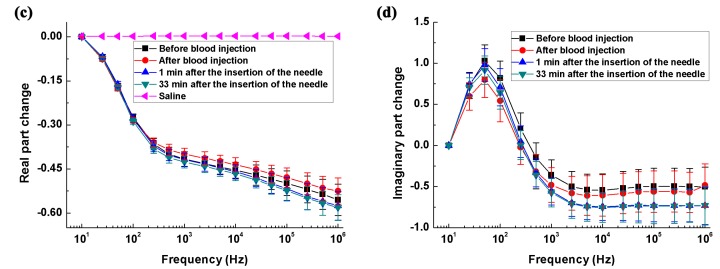
The brain impedance spectra of the intracerebral hemorrhage group and its control group. (**a**,**b**) Real and imaginary parts of brain impedance spectra; (**c**,**d**) The changes in the real and imaginary parts of brain impedance relative to the impedance at 10 Hz.

As shown in Figure 3c, relative to the impedance at 10 Hz, the impedance change of the saline solution remained at nearly zero across the whole frequency range, indicating the reliability of our measurements.

Figure 3a,b shows the real and imaginary parts of impedance spectra of the hemorrhage group and its control. From 10 Hz to 1 kHz, the real part of brain impedance decreased greatly and there was a local extreme point near 50 Hz for imaginary part. According to Schwan and Morowitz’s dispersion theory [29], 50 Hz should correspond to the characteristic frequency of initial dispersion. Figure 3c,d shows the changes in real and imaginary parts of brain impedance relative to the impedance at 10 Hz. Regarding the real part of brain impedance, there was a steep and nearly linear decrease (logarithmic frequency) of approximately 35%, from 10 Hz to 200 Hz. Between 200 Hz and 1 MHz, the real part decreased slowly from −35 to −60%. The imaginary part of brain impedance initially increased from 10 Hz to 100 Hz (a change of approximately 100%), then decreased within 100 Hz–1 kHz (a change of 150%), and finally remained constant between 2 kHz and 1 MHz.

**Figure 4:**
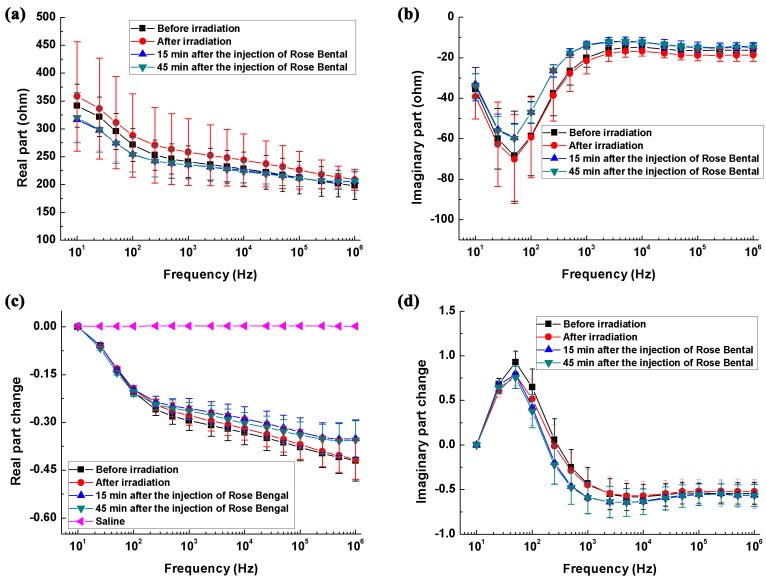
The brain impedance spectra of the ischemia group and its control. (**a**,**b**) The real and imaginary parts of the impedance spectra; (**c**,**d**) The changes in the real and imaginary parts of brain impedance relative to the impedance at 10 Hz.

Figure 4a,b shows the real and imaginary parts of the brain impedance spectra of the ischemia group and its control. The magnitude of real and imaginary parts after ischemic stroke was greater than that recorded before stroke. Figure 4c,d shows the changes in the real and imaginary parts of the brain impedance spectra relative to the impedance at 10 Hz. The magnitude and trend of changes in the real and imaginary parts for the ischemia group and its control were like those for the hemorrhage group and its control.

### 3.2. Difference in Impedance Spectra between Healthy and Stroke-Affected Brains (Hemorrhagic or Ischemic Brain)

**Figure 5:**
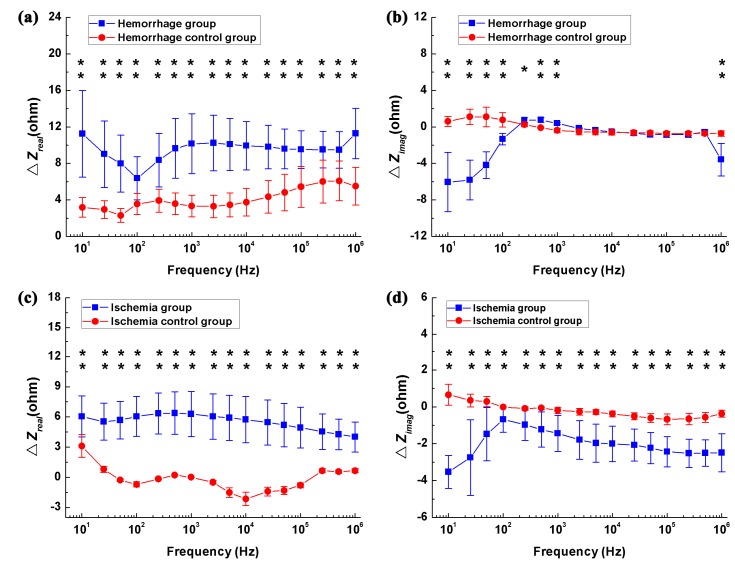
The change in the brain impedance spectra before and after stroke in relation to the control groups. (**a**,**b**) Real and imaginary parts of brain impedance differed in the hemorrhage group (measured before and after blood was injected) and its control (measured 1 min and 33 min after the needle was inserted); (**c**,**d**) Real and imaginary parts of brain impedance differed in the ischemia group (measured before and after irradiation with green cold light) and its control group (measured 15 min and 45 min after injection of rose bengal dye). ΔZ denotes the brain impedance change between the brain impedance ($Z^{A}$ and $Z^{B}$) measured at two time points (A and B, if A and B represent the two time points), i.e., $\Delta Z=Z^{B}-Z^{A}$. * *p* < 0.05, ** *p* < 0.01.

Figure 5a,b shows the differences in real and imaginary parts of brain impedance spectra in the hemorrhagic group (measured before and after blood was injected) and its control (measured 1 min and 33 min after the needle was inserted). As shown in Figure 5a, the difference in the real part before and after blood was injected decreased initially from 10 Hz to 100 Hz and then increased within 100 Hz–1 MHz, varying in the range from 6.4 ohms (smallest value at 100 Hz) to 11.3 ohms (largest value at 1 MHz). In contrast, the difference in the real part was less than six ohms in the hemorrhage control group. Across the whole frequency range, there were significant differences in the real part between the hemorrhage group and its control (*p* < 0.01). In the hemorrhage group, the difference in the imaginary part increased sharply by 5.3 ohms from 10 Hz to 500 Hz, declined slowly by one ohm within 500 Hz–200 kHz and finally increased dramatically by three ohms between 500 kHz and 1 MHz. In contrast, the difference in the imaginary part was less than two ohms in the hemorrhage control group. Across the 10 Hz–1 kHz and 5 kHz–1 MHz frequency ranges, the difference in the imaginary part differed significantly between the hemorrhage group and its control (*p* < 0.05). These results indicated that brain impedance spectra changed significantly following hemorrhagic stroke.

Figure 5c,d shows the differences in real and imaginary parts of brain impedance spectra in the ischemia group (measured before and after irradiation with green cold light) and its control group (measured 15 min and 45 min after the injection of rose bengal dye). In Figure 5c, the difference in the real part before and after irradiation with green cold light changed slowly from 10 Hz to 500 Hz and then decreased significantly from 500 Hz to 1 MHz, with difference greater than four ohms across the whole frequency range. The difference in the real part in the hemorrhage group was significantly larger than those in its control group (*p* < 0.01). The difference in the imaginary part increased quickly from 10 Hz to 100 Hz, then decreased quickly between 100 Hz and 1 MHz. Across the whole frequency range, the change in the imaginary part differed between the hemorrhage group and its control (*p* < 0.01). These results suggest that a significant change in brain impedance spectra was caused by ischemic stroke.

### 3.3. Difference in the Rate of Change in Brain Impedance with Frequency between Ischemic and Hemorrhagic Stroke

**Figure 6:**
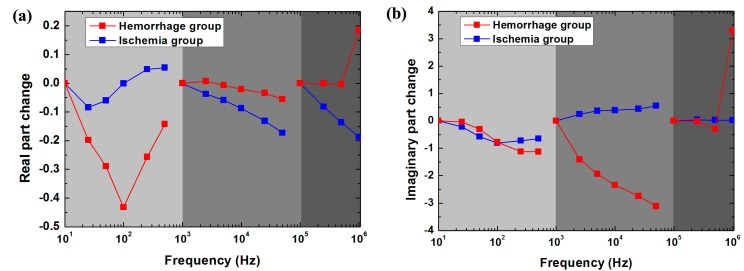
The rate of change in the real (**a**) and imaginary (**b**) parts of brain impedance after hemorrhagic and ischemic stroke in the low (10 Hz–1 kHz; reference frequency: 10 Hz), intermediate (1–100 kHz; reference frequency: 1 kHz) and high frequency (100 kHz–1 MHz; reference frequency: 100 kHz) ranges, as obtained from $\Delta Z=\Delta Z_{real}+j\Delta Z_{imag}=Z_{after}-Z_{before}$, where $Z_{before}$ and $Z_{after}$ represent brain electrical impedance before and after stroke, respectively.

As shown in Figure 6a, within 10 Hz–1 kHz, the change in the real part of brain impedance after hemorrhage decreased by up to approximately 45% from 10 Hz to 100 Hz and then increased rapidly. In contrast, the range of variation remained small (<5%) after ischemia. Between 1 kHz and 100 kHz, the magnitude of the change in the real part after hemorrhage (5%) was greater than that after ischemia (20%). From 100 kHz to 1 MHz, the change in the real part after ischemia decreased with frequency (approximately 5%), whereas the change after hemorrhage was almost zero within 100 kHz–500 kHz but increased significantly by approximately 20%within 500 kHz–1 MHz. Thus, across the whole frequency range, the rate of change in the real part was much different between hemorrhagic and ischemic stroke.

As shown in Figure 6b, from 10 Hz to 1 kHz, the change in the imaginary part of brain impedance decreased with frequency after both hemorrhage and ischemia. Within 1 kHz–100 kHz, the change in the imaginary part after hemorrhage decreased with frequency (threefold), whereas it increased with frequency after ischemia (0.75-fold). Between 100 kHz and 1 MHz, the change in the imaginary part remained nearly zero after both hemorrhage and ischemia. Across the range from 100 kHz to 500 kHz, the change in the imaginary part was very small (<0.4-fold) after hemorrhage. However, the change after hemorrhage was far greater (3.5-fold) than that after ischemia from 500 kHz to 1 MHz. Therefore, the rate of change in the imaginary part differed greatly between hemorrhagic and ischemic stroke within 1 kHz–100 kHz and within 500 kHz–1 MHz.

### 3.4. Histopathology

**Figure 7:**
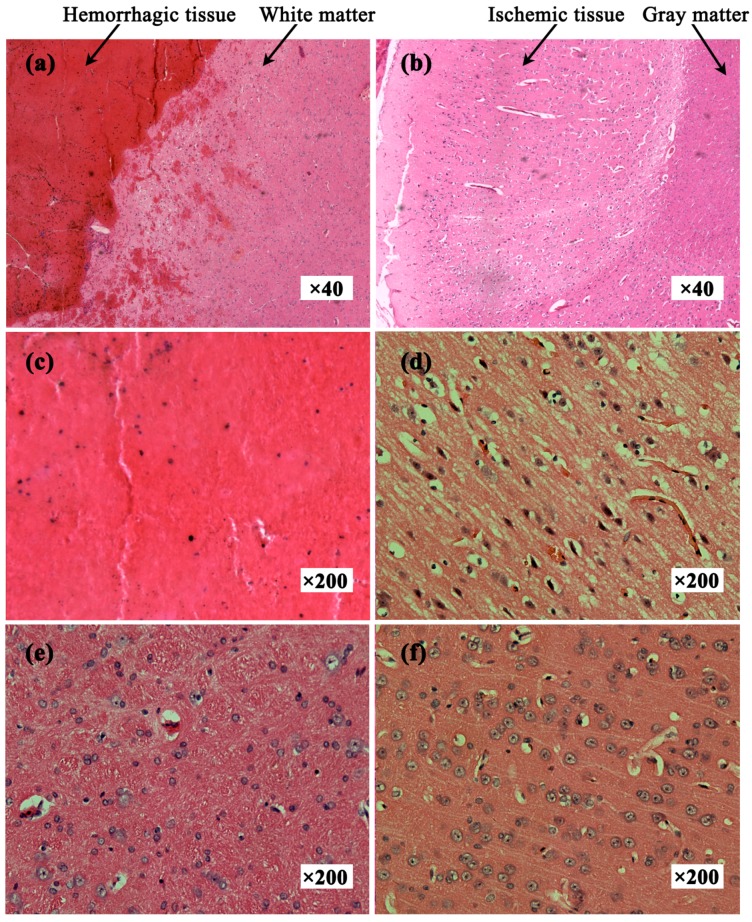
Hematoxylin and eosin-stained tissue sections. (**a**) Hemorrhagic tissue with surrounding white matter; (**b**) Ischemic tissue with surrounding gray matter; (**c**) Hemorrhagic tissue (blood clot); (**d**) Ischemic tissue; (**e**) Normal white matter; (**f**) Normal gray matter.

Figure 7a shows the hemorrhagic tissue (blood clot) and surrounding normal white matter. The boundary between blood and white matter was clear and some blood penetrated the white matter. Figure 7b shows the ischemic tissue and surrounding normal gray matter. Distinct colors clearly delineated ischemic tissue and gray matter. Many red cell nuclei could be observed in the hemorrhagic tissue (Figure 7c) and the normal white matter cells were arranged densely and neatly (Figure 7e). Compared with normal gray matter cells (Figure 7f), ischemic tissue cells were enlarged, suggesting edema secondary to ischemia (Figure 7d). Thus, hemorrhagic tissue, ischemic tissue, white matter and gray matter showed different histological features.

## 4. Discussion

### 4.1. Summary of the Results and Comparative Analysis of the Literature

In this study, we established localized intracerebal hemorrhage and ischemic models in rabbits, and comprehensively measured and analyzed the impedance spectra of healthy, hemorrhagic and ischemic brain from 10 Hz to 1 MHz.

#### 4.1.1. The Impedance Spectra of the Healthy Brain

As for the impedance spectra of the healthy brain, the change in the real part of brain impedance was approximately 30% (25% for the ischemia control group in Figure 3c and 35% for the hemorrhage control group in Figure 4c) from 10 Hz to 200 Hz. Within 200 Hz–1 MHz, this ranged from 30 to 60%. Our results were comparable to those reported by Ranck (30% within 5 Hz–5 kHz) [15], Lingwood et al. (35% within 10 Hz–5 kHz) [14] and Dowrick et al. (40% within 10 Hz–3 kHz) [16]. In terms of measured phase, Lingwood et al. [14] and Dowrick et al. [16] measured a phase angle of less than one degree below 5 kHz, while we reported that the real part of brain impedance was nearly 10 times that of the imaginary part, i.e., the phase angle was approximately 6 degrees. Our reports were supported by Ranck who documented a phase angle of seven degrees [15]. The explanation for the different phase angles is not yet known.

#### 4.1.2. The Impedance Spectra of the Hemorrhagic Brain

Across the 10 Hz–1 MHz range, brain impedance was higher after blood was injected (hemorrhagic brain) than that before (healthy brain) (Figure 5a). The results are in agreement with those of Dai et al. [23], who established a hemorrhagic model by injecting autologous blood into the subarachnoid space, and found that brain impedance increased after blood was injected (60 min after completion of blood injection, at 50 kHz). However, Manwaring et al. [24] and Dowrick et al. [20] established brain hemorrhage models in piglets by injecting autologous blood, and found that brain impedance decreased at 50 kHz after blood was injected. The reason for the difference is not clear.

**Figure 8:**
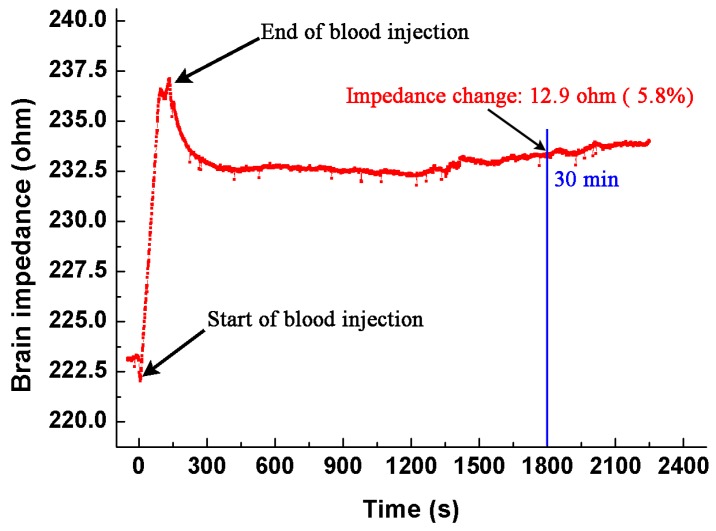
The brain impedance during the whole procedure (from the start of blood injection to 30 min afterwards).

To further validate our results, we carried out an experiment using an animal model. The method to establish the intracranial hemorrhage model was the same as that described in Section 2.3. During the whole procedure of blood injection and 30 min after injection, we monitored the brain impedance using the EIT system developed by our group, which could operate from 1 kHz to 190 kHz, with precision better than 0.05% [30]. The current used was 200 μA at 50 kHz. Figure 8 shows the brain impedance during the entire experiment. In the procedure of blood injection, the brain impedance increased sharply. After the completion of injection, the brain impedance decreased slowly with little change in the end. The change of brain impedance before and 30 min after blood injection is 12.9 Ω, which is approximate to the result (10.5 Ω) we reported.

Intuitively, the brain impedance should decrease because the impedance of fresh blood is lower than that of brain. However, our results suggest otherwise. There are two possible reasons that may account for the discrepancies. First, the space-occupying effect due to the hemorrhagic lesion may be a main factor that caused a non-negligible brain impedance change. In practice, when the blood is injected into brain, normal brain tissues at the injection site will be squeezed to the surroundings. According to Monro-Kellie’s hypothesis [31], the skull-forming cavity can be considered as a fixed-volume container, thus the change of the volume and location of the intracranial tissues will lead to the change of brain impedance [23]. During blood injection, the squeezed brain tissue may affect the distribution of intracranial cerebrospinal fluid (CSF). We presume that the volume of intracranial CSF may decrease because the hemorrhagic tissue (injected blood) occupies a certain intracranial volume. Since the impedance of CSF is much lower than that of the hemorrhagic tissue (clotted or non-clotted blood) and brain tissues (Table 1), the brain impedance increases as a result. Dai et al. [23] found that an increase of impedance in all region except the site of blood injection, and presumed that the change of the CSF distribution might have an influence on the brain impedance.

Second, normal brain tissues surrounded by the hemorrhagic tissue underwent a pathological change, which may be another key factor that affected the brain impedance. As shown in Figure 7a, the normal tissue cells surrounding the hemorrhagic tissue were enlarged and exhibited ischemic pathological changes. The reason for this may be that the hemorrhagic tissue squeezed the normal tissues, resulting in a significant reduction of blood supply to those normal tissues.

#### 4.1.3. The Impedance Spectra of the Ischemic Brain

In the case of the impedance spectra of the ischemic brain, taking 10 Hz as the reference frequency, the real part of brain impedance changed by 30.7% in the ischemic brain and by 32.1% in the healthy brain at 5 kHz (Figure 4c). Hence, below 5 kHz, the real part of brain impedance changed less with frequency after ischemia than before it. Our results are in agreement with Dowrick et al. (30% in the ischemic brain and 40% in the healthy brain within 10 Hz–5kHz) [16]. Seoane et al. established a brain hypoxia/ischemia model in piglets by reducing oxygen intake, and measured the brain impedance before and after hypoxia from 20 kHz to 750 kHz [18]. They reported that the brain impedance increased following ischemia, which is consistent with the findings in this study (Figure 5c). Nevertheless, in their study, at 750 kHz, the brain impedance decreased following ischemia, which is contrary to the results of our study. This was probably because the different methods were used to establish the ischemic model. During photothrombotic stroke method, the fluorescent agent produces free radicals under light irradiation, and free radicals damage vascular endothelial cells. Blood platelets adhere to the damaged endothelial cells, triggering release reactions and subsequent intravascular coagulation. As clots form, blood flow to the brain is impaired; this causes tissue hypoxia and blocks blood flow [33]. In contrast, when brain hypoxia is simulated by reducing oxygen intake, blood still flows into the brain although it contains less oxygen.

### 4.2. Optimal Frequency Ranges for Stroke Detection Using MFEIT

Optimal frequency ranges are one of the key issues when considering MFEIT to detect stroke, because the data obtained across the optimal frequency ranges may include additional information useful to detect stroke.

In MFEIT, the first objective is to detect stroke, i.e., to differentiate a healthy brain from a stroke-affected one. Following hemorrhagic stroke, the difference in the real part of brain impedance (measured before and after hemorrhagic stroke) could be up to 11 Ω above 1 kHz and the difference in the imaginary part was greater than 3.5 Ω below 1 kHz or above 500 kHz (Figure 5b). Hence, the real part of brain impedance across the 1 kHz–1 MHz range or the imaginary part of brain impedance across the 10 Hz–1 kHz and 500 kHz–1 MHz ranges may help differentiate a healthy brain from a hemorrhagic one. Following ischemic stroke, the difference in the real part of brain impedance (measured before and after ischemic stroke) did not change significantly with frequency and the magnitude of change was approximately five ohms (Figure 5c). Additionally, the difference in the imaginary part of brain impedance was greater than two ohms above 10 kHz (Figure 5d). Hence, the real part of brain impedance across the whole frequency range or the imaginary part of brain impedance above 10 kHz may help differentiate a healthy brain from an ischemic one.

Another key component to bear in mind when considering MFEIT to detect stroke is differentiating stroke subtypes (i.e., ischemic vs. hemorrhagic). Figure 6a shows the different rates of change in the real part of brain impedance after hemorrhagic and ischemic stroke across the whole frequency range. In particular, from 500 kHz to 1 MHz, the change in the real part of brain impedance after hemorrhagic stroke was significantly greater than that after ischemic stroke (20% vs. approximately 5%). Across the intermediate frequency range, the change in the imaginary part of brain impedance after hemorrhagic stroke was significantly greater than that after ischemic stroke (threefold vs. 0.75-fold). Moreover, within 500 kHz to 1 MHz, the change in the imaginary part of brain impedance differed significantly between after hemorrhagic stroke and after ischemic stroke (3.5-fold vs. 0.4-fold). Hence, the intermediate frequency range (1 kHz–100 kHz) and the 500 kHz–1 MHz frequency range may be the optimal ranges to differentiate stroke subtypes.

In this study, the purpose of comparing the difference between brain impedance spectra caused by ischemic and hemorrhagic stroke respectively (in Section 3.3) is to find the optimal frequency range for stroke detection, in which the differences resulted from the two types of stroke prove to be specific. In practice, because the measured brain impedance spectra include both the information of the stroke lesion and normal head tissues, it is impossible to directly employ the brain impedance spectra after stroke (even in the optimal frequency range) to detect the disease. However, according to our results of the comparison, the brain impedance spectra in the optimal frequency range may provide helpful information for stroke detection using MFEIT.

### 4.3. Limitaions of This Study

In this study, we measured the brain impedance spectra 30 min after stroke to compare it with the brain impedance before stroke. Our results indicated that the brain impedance spectra significantly changed because of the occurrence of stroke and the changes in brain impedance spectra caused by ischemic and hemorrhagic stroke respectively also varied from each other. These results further validated the feasibility of stroke detection as well as stroke type differentiation using MFEIT. However, because the ischemic stroke patients need treatment within 3–4.5 h after the onset of stroke in practice, it is also important to measure the change of brain impedance spectra taking place in the period of 30–90 min after stroke. In the future, we will conduct relevant investigation regarding the change in brain impedance spectra over time after stroke.

## 5. Conclusions

In this study, we established the intracerebral hemorrhage and ischemic models in rabbits, and comprehensively measured and compared the brain impedance spectra of healthy, hemorrhagic and ischemic brain at frequencies from 10 Hz to 1 MHz. The results demonstrated that the impedance spectra of stroke-affected (hemorrhagic and ischemic) brain significantly differed from healthy brain (within the 10 Hz–1 MHz range, the real part of brain impedance differed by greater than six ohms between healthy and stroke-affected brain); the rate of brain impedance in hemorrhagic and ischemic brain with regard to frequency was distinct (within the 500 kHz–1 MHz range, the change of the imaginary part of brain impedance in hemorrhagic brain was almost three times that of ischemic brain). Data analysis also demonstrated that the real part of brain impedance across the 1 kHz–1 MHz range or the imaginary part of brain impedance across the 10 Hz–1 kHz and 500 kHz–1 MHz ranges may help differentiate a healthy brain from a hemorrhagic one; the real part of brain impedance across the whole frequency range, or the imaginary part at frequencies above 10 kHz, may help differentiate an ischemic brain from a healthy one; the 1 kHz–100 kHz and 500 kHz–1 MHz ranges may be the optimal frequency ranges to detect stroke subtypes. These findings further confirm the feasibility of using MFEIT to detect stroke and provide valuable data for continuing research in this field.

## Figures and Tables

**Table 1:** The conductivity of different brain tissues at 50 kHz [8,32].

Tissues	Conductivity (S/m)
White matter	0.1183
Grey matter	0.17281
Cerebrospinal fluid (CSF)	1.8
Hemorrhagic tissue (clotted blood)	0.07942
Fresh blood	0.7
